# Transcriptomics and biochemical evidence of trigonelline ameliorating learning and memory decline in the senescence-accelerated mouse prone 8 (SAMP8) model by suppressing proinflammatory cytokines and elevating neurotransmitter release

**DOI:** 10.1007/s11357-023-00919-x

**Published:** 2023-09-18

**Authors:** Sharmin Aktar, Farhana Ferdousi, Shinji Kondo, Tamami Kagawa, Hiroko Isoda

**Affiliations:** 1 Alliance for Research on the Mediterranean and North Africa (ARENA), University of Tsukuba, Tsukuba, Japan; 2 Institute of Life and Environmental Sciences, University of Tsukuba, Tsukuba, Japan; 3 DyDo DRINCO, INC, Osaka, Japan; 4 Institute of Life and Environmental Sciences, University of Tsukuba, 1-1-1 Tennodai, Tsukuba, Ibarak 305-8572 Japan

**Keywords:** Trigonelline, Neurotransmitter, Neuroinflammation, Proinflammatory cytokine, DNA Microarray, Cognitive function, SAMP8

## Abstract

In recent years, exploring natural compounds with functional properties to ameliorate aging-associated cognitive decline has become a research priority to ensure healthy aging. In the present study, we investigated the effects of Trigonelline (TG), a plant alkaloid, on memory and spatial learning in 16-week-old senescence-accelerated mouse model SAMP8 using an integrated approach for cognitive and molecular biology aspects. After 30 days of oral administration of TG at the dose of 5 mg/kg/day, the mice were trained in Morris Water Maze task. TG-treated SAMP8 mice exhibited significant improvement in the parameters of escape latency, distance moved, and annulus crossing index. Next, we performed a whole-genome transcriptome profiling of the mouse hippocampus using microarrays. Gene ontology analyses showed that a wide range of biological processes, including nervous system development, mitochondrial function, ATP synthesis, and several signaling pathways related to inflammation, autophagy, and neurotransmitter release, were significantly enriched in TG-treated SAMP8 compared to nontreated. Further, a nonlinear dimensionality reduction technique, Uniform Manifold Approximation and Projection (UMAP), was applied to identify clusters of functions that revealed TG primarily regulated pathways related to inflammation, followed by those involved in neurotransmitter release. In addition, a protein–protein interaction network analysis indicated that TG may exert its biological effects through negatively modulating Traf6-mediated NF-κB activation. Finally, ELISA test showed that TG treatment significantly decreased proinflammatory cytokines- TNFα and IL6 and increased neurotransmitters- dopamine, noradrenaline, and serotonin in mouse hippocampus. Altogether, our integrated bio-cognitive approach highlights the potential of TG in alleviating age-related memory and spatial impairment.

**Supplementary Information:**

The online version contains supplementary material available at 10.1007/s11357-023-00919-x.

## Introduction

Cognitive aging refers to age-related impairments in cognitive functions, including slower problem-solving, reduced fluid reasoning, diminished perceptual speed, and impaired memory and spatial ability. It stands as one of the most common health concerns among older individuals [1]. It is estimated that cognitive impairment affects over 11.7% of adults aged 65 and above, with the prevalence increasing to approximately 40% among those aged 80 and older [2]. According to the World Health Organization (WHO), neurodegenerative disorders will rank second among all causes of natural mortality by 2040 [3]. The rise in life expectancy worldwide, particularly in advanced nations, contributes to a substantial increase in the global incidence of cognitive impairment. Cognitive aging significantly impacts the quality of life for older adults, places strain on healthcare providers and systems and imposes substantial economic burdens on families and society as a whole [4]. Therefore, it is imperative to take proactive measures to support brain health among older adults and foster healthy aging on a broader scale.

Since the inception of cognitive aging theory in the mid-1960s, technological advancements have provided us with a better understanding of the neural mechanisms underlying cognitive aging. One common phenomenon of cognitive aging is neuroinflammation, characterized by an elevation in proinflammatory cytokines in the brain, including tumor necrosis factor-alpha (TNFα) and interleukin 6 (IL6) [5, 6]. Neuroinflammation not only leads to damage and dysfunction of neurons and other brain cells but also disrupts the balance of neurotransmitters such as dopamine (DA), noradrenaline (NA), serotonin (5-HT), and gamma-aminobutyric acid (GABA) in the brain, resulting in impaired transmission of signals and cognitive processes [5, 7]. Therefore, a growing focus is on addressing neuroinflammation and restoring neurotransmitter balance as key research areas to alleviate cognitive decline and support healthy cognitive aging [8, 9].

In recent years, there has been growing evidence that certain functional foods, beverages, and natural bioactive compounds possess neuroprotective properties and can effectively slow the progression of brain aging [10, 11]. These compounds have demonstrated various benefits, including reduction of neuroinflammation, restoration of neurotransmitter balance, and promotion of neuronal differentiation, all while exhibiting minimal adverse effects [12, 13]. However, it is crucial to comprehend how these potential compounds affect intercellular signaling molecules and intracellular signal transduction pathways, ultimately increasing the likelihood of healthy aging.

In this regard, trigonelline (TG), a naturally occurring alkaloid compound (N-methyl nicotinic acid) found in various plants, including coffee beans, fenugreek, and Japanese radish, has been extensively studied for its numerous biological activities, including antimicrobial, anticancer, antidiabetic, antihypertensive, and anti-hyperlipidemic effects [14–16]. Notably, previous studies also reported that TG promotes the regeneration of dendrites and axons in cortical neurons and inhibits the formation of advanced glycation end products in vitro, and improves learning and memory impairment in the in vivo models of Alzheimer’s disease and neuroinflammation [17–19]. However, the effects of TG on age-related cognitive decline and the underlying molecular mechanisms remain to be fully investigated.

In the present study, we aimed to investigate the potential of TG in mitigating the age-related decline in memory and cognition using a mouse model of accelerated aging, the senescence-accelerated mouse-prone 8 (SAMP8) mice, with senescence-accelerated mouse-resistant 1 (SAMR1) mice as the control group. The SAMP8 mouse is a naturally occurring animal model that exhibits an accelerated aging phenotype characterized by a progressive cognitive decline as well as neurodegenerative changes [20–22]. Despite the complex nature of aging, which involves the gradual deterioration of cognitive, physical, and biological functions at different rates, previous studies have demonstrated cognitive impairments in younger SAMP8 mice across a variety of behavioral tasks as early as four months of age [23]. We assessed the effects of chronic administration of TG over a 30-day period on spatial memory deficits in 16-week-old SAMP8 mice using the Morris water maze (MWM) test, a widely used and reliable hippocampus-dependent memory task. Furthermore, we conducted a comprehensive whole-genome transcriptomics analysis in the mice hippocampus, employing an untargeted approach to uncover the biological events triggered by TG supplementation and elucidate the underlying mechanisms involved.

## Materials and Methods

### Experimental Animals 

For our in vivo investigations, we utilized 16-week-old male SAMP8 mice (Japan SLC, Shizuoka, Japan). These mice exhibit spatial learning deficits and symptoms of memory loss that become apparent from the age of 16 weeks [24]. To serve as a control group representing normal aging, we used senescence-accelerated mouse resistant-1 (SAMR1) mice, which possess a genotype resistant to senescence and exhibit a normal aging phenotype.

The mice were housed individually in cages maintained at a temperature range of 21–23 °C. The light–dark cycle followed a photoperiod of 12 h of light and 12 h of darkness. They were provided with ad libitum access to food and water. All animal procedures were conducted in accordance with the guidelines set forth by the Physiological Society of Japan Council. The experimental protocols for this study were approved by the University of Tsukuba Ethics Animal Care Committee, ensuring strict adherence to ethical considerations.

### TG preparation and oral administration

Following a one-week acclimatization period, the SAMP8 mice were randomly divided into two groups: one group received water administration as a placebo (n = 8), while the other group received oral administration of TG (n = 6–8). Similarly, the SAMR1 mice, serving as the control group for normal aging, also received water administration (n = 8).

Commercially available trigonelline chloride (TG), obtained from Cayman Chemical (Tokyo, Japan, catalog no: 11904), was used in the study. It was dissolved in Milli-Q water. Prior to the administration in mice, the cytotoxicity of TG was assessed in human neurotypic SH-SY5Y cells using the MTT assay (Fig. S1). Concentrations of TG ranging from 5 to 80 µM were tested in the MTT assay. It was observed that increasing concentrations of TG did not result in cell death; instead, they promoted cell proliferation and viability (Fig. S1).

The SAMP8 mice were orally administered TG at a dose of 5 mg/kg/day for a duration of 30 days. To facilitate the oral administration, a sterile feeding needle with a length of 1.2 inches and a diameter of 1.25 mm, featuring a ball-shaped opening, was utilized. No anesthesia was required for the administration process. The concentration and dosage of TG were determined based on the results obtained from the MTT assay/ previous investigations (Supplementary Fig. S1).

**Fig. 1:**
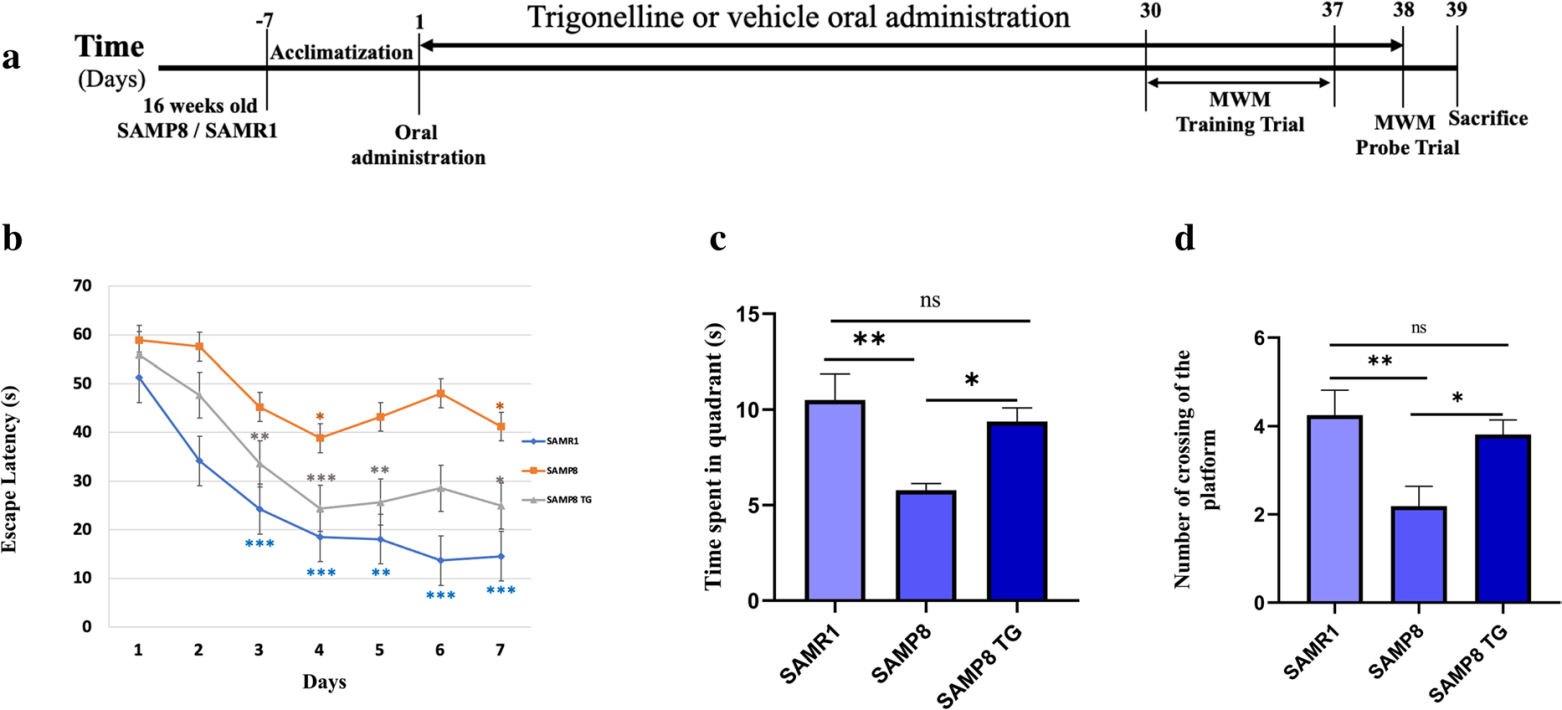
Effects of TG on spatial learning and memory examined with the Morris water maze. **a** Experimental timeline. **b** Escape latency of each treatment group at different time points. The asterisk refers to the within-group significant difference compared to Day 1. The differences among treatment groups were assessed using two-way repeated-measures ANOVA followed by Dunnett’s post hoc test. *P < 0.05, **P < 0.01, ***P < 0.001 **(c)** Effects of TG on time spent in the target quadrant in the probe test, A comparison was performed by one-way ANOVA followed by Dunnett’s post hoc *P < 0.05, **P < .001; ns, non-significant. **d** Effects of TG on the platform crossing number in the probe test. Comparisons between the groups were performed using one-way ANOVA followed by Fisher’s LSD post hoc test: *P < 0.05, **P < 0.01; ns, non-significant. Values are presented as mean ± *SEM (n* = *6* ~ *8 animals per group)*

Upon completion of the 30-day treatment period, the mice underwent a behavioral test that spanned an additional eight days. Throughout this period, the treated group continued to receive the TG compound via oral administration (Fig. 1a).

### Morris water maze test (MWM) 

The MWM test is a robust and reliable method closely linked to hippocampal synaptic plasticity. It is widely used to evaluate rodents' spatial learning and memory [25]. During the MWM test, the animals are placed in a pool of water with a submerged platform. The time taken by the mice to locate the platform and escape the maze, known as escape latency, serves as a reliable indicator of the learning process.

We used a circular pool measuring 120 cm in diameter, 45 cm in height, and 30 cm in depth, maintaining a pool temperature of 23 ± 2 C. Temporary boundaries were set to define the four quadrants of the pool. An escape platform with a diameter of 10 cm was positioned 1 cm below the water's surface in the center of one of the quadrants. The mice were given a maximum of sixty seconds to swim and locate the platform during each of the four daily trials conducted over a period of seven days.

On the final day, the escape platform was removed, and a probe test was conducted, allowing the mice to swim freely for sixty seconds. During this test, the time spent in the target quadrant and the number of crossings over the previous platform location were recorded as measures of spatial memory performance.

### Enzyme-linked immunosorbent assay (ELISA)

The mice were euthanized through quick cervical dislocation, and whole brain samples were collected. Subsequently, the brain samples were carefully dissected to extract the hippocampus. The isolated hippocampus was subsequently homogenized using ultrasonication in RIPA lysis buffer (Santa Cruz, Dallas, Texas, United States) supplemented with protease inhibitors. The homogenates were then centrifuged at 16,000 × G for 20 min, and the resulting supernatant was collected for subsequent analysis.

The levels of neurotransmitters were assessed using an ELISA kit (ImmSmol, Talence, France). Specifically, the neurotransmitters DA (immuSmol BA E 5300R), NA (immuSmol BA E-5200R) and 5-HT (immuSmol BA E-5900R) were quantified. Additionally, the levels of brain-derived neurotrophic factor (BDNF) (ImmSmol, Talence, France; Proteintech KE00096) and proinflammatory cytokines TNFα and IL6 were measured using an ELISA kit (BioVison, Milpitas, California, United States), following the manufacturer's instructions. A 2D Quant kit (GE Healthcare and Piscataway, New Jersey, United States) was used to standardize the levels of each neurotransmitter relative to the total protein concentration.

### RNA isolation and quantification

Total RNA was extracted from the hippocampus using the ISOGEN kit (Nippon Gene Co. Ltd., Tokyo, Japan) according to the manufacturer's instruction. The NanoDrop 2000 spectrophotometer (Thermo Fisher Scientific, Wilmington, DE, USA) was used to measure the RNA concentration.

### Microarray experiment

DNA microarray analysis was conducted on three randomly selected hippocampal samples from each group (SAMR1, SAMP8, and TG-treated SAMP8). The GeneChip WT PLUS Reagent Kit (ThermoFisher Scientific, Waltham, MA, USA) and GeneChip™ Hybridization, Wash and Stain Kit (ThermoFisher Scientific, Waltham, MA, USA) were utilized following the manufacturer's instructions.

Briefly, complementary DNA (cDNA) was synthesized from 100 ng of RNA solutions. Subsequently, cRNA was generated through in vitro transcription of the cDNA and underwent purification and reverse transcription. The resulting single-stranded cDNA (ss-cDNA) was then synthesized, purified, fragmented, and labeled as per the manufacturer's guidelines.

The Clariom S array Mouse (ThermoFisher Scientific, Waltham, MA, USA) was hybridized on the GeneChip™ Fluidics Station (ThermoFisher Scientific, Waltham, MA, USA). The arrays were then scanned using the GeneChip Scanner (ThermoFisher Scientific, Waltham, MA, USA). And, finally the raw signal intensity values (CEL files) were computed from the scanned array images.

### Microarray data processing and analysis

The raw image data obtained from scanning were subjected to standardization using the signal space transformation robust multi-chip analysis (SST-RMA) algorithm in the Transcriptome Analysis Console (TAC) software (version 4.0.2, ThermoFisher Scientific, Waltham, MA, USA). A One-way ANOVA followed by an empirical Bayes correction was performed to analyze differential expression. Genes that met the filter criteria of a P value < 0.05 (one-way between-subject ANOVA) and a fold change (FC) > 1.1 (in linear space) were considered as differentially expressed genes (DEGs).

Gene ontology (GO) enrichment analysis was conducted using the Database for Annotation, Visualization, and Integrated Discovery (DAVID) web tool (http://david.ncifcrf.govt/) [26]. Terms with P value < 0.05 (modified Fisher’s Exact Test) were considered significantly enriched. The interactions between GO terms were visualized using the REVIGO tool (http://revigo.irb.hr/) [27]. The color of the disc represents the significance of each GO term, while the size of the disc corresponds to the number of genes in that category. The thickness of the grey lines indicates the level of simRel semantic similarity between the categories. The spatial arrangement of the discs also reflects the grouping of categories based on their semantic similarity.

The scatterplot depicting clusters of gene sets with similar functional pathways was generated using the Enrichr Appyter visualization tool (https://maayanlab.cloud/Enrichr/) [28]. The NCATS BioPlanet 2019 library was utilized to identify clusters of gene set [29]. Term frequency–inverse document frequency (TF–IDF) values were calculated for each gene set term, and the resulting values were subjected to dimensionality reduction using the Uniform Manifold Approximation and Projection (UMAP) technique. The terms are plotted based on the first two UMAP dimensions, with each term being assigned a color corresponding to the automatically identified clusters determined through the Leiden algorithm applied to the TF–IDF values.

SynGO web tool was used to explore overrepresented synaptic GO terms and to generate the sunburst plots (https://www.syngoportal.org/). SynGO is an open-access knowledgebase dedicated to synapse research, offering approximately 3000 annotations related to synapse-specific protein location or function and around 1100 distinct genes/proteins [30]. Heatmaps were generated using an online tool Morpheus (https://software.broadinstitute.org/morpheus).

An online tool, Network Analyst version 3.0, was employed to construct the protein–protein interaction (PPI) networks [31]. The first-order PPI network was built based on the IMEx Interactome database, which consists of comprehensive, literature-curated data sourced from Innate DB. Heat maps were generated using Morpheus online tool [32].

### Statistical analysis

The values are reported as the mean ± SEM. To compare escape latency, repeated-measure two-way analysis of variance (ANOVA) with Dunnett's post hoc test was employed. A one-way ANOVA followed by Fisher's LSD test was used to identify statistically significant differences in other behavioral tests and the expressions of proteins. The normality of continuous variables of ELISA was assessed using the Shapiro–Wilk normality test. Statistical analysis was performed using the GraphPad Prism 8 software (GraphPad, San Diego, CA, United States). A *P* value < 0.05 was considered statistically significant.

## Results

### TG improved the spatial learning and memory of the SAMP8 mice in the MWM test

We used the hippocampus-dependent memory task MWM to evaluate the effects of TG administration on spatial learning and memory in SAMP8 mice. On the first day of the training trial in the MWM test, there were no significant differences in escape latency within the groups (Fig. 1b). However, as the training sessions progressed, the SAMR1 mice exhibited a significant decrease in escape latency starting from the second day. In contrast, the SAMP8 group did not show consistent changes in latency compared to their first day's performance. Still, their latency was comparatively higher than that of the SAMR1 group from the second day onwards. Similarly, the TG-treated SAMP8 group exhibited a significant decrease in escape latency from the second day of testing within the group (Fig. 1b).

In the probe trial on the 8th day, the SAMP8 mice had a significant decrease compared with the SAMR1 mice in time spent in the target quadrant (Δmean = 3.6; *P* < 0.05) and crossing number (Δmean = 1.6 ± 0.6; *P* < 0.01). However, TG treatment significantly increased the time spent in the target quadrant for the SAMP8 mice (Δmean = 3.5 s; *P* < 0.05) as well as the crossing number (Δmean = 1.6; *P* < 0.05) when compared to the untreated SAMP8 mice.TG treatment significantly increased the time spent in the target quadrant (Δmean = 3.5 s; *P* < 0.05) and crossing number (Δmean = 1.6; *P* < 0.05) compared to the nontreated SAMP8 mice (Fig. 1c,d).

### TG treatment significantly regulated gene expressions in mice hippocampus

Dynamic alterations in gene transcription are crucial for establishing long-term memory and memory retrieval processes in the brain. Given our observation that TG treatment significantly improved the hippocampal-dependent spatial memory decline in SAMP8 mice, our subsequent objective was to investigate the potential biological effects of TG in the hippocampus. Therefore, we conducted an untargeted analysis of whole-genome transcriptomics in the hippocampus of mice.

**Fig. 2:**
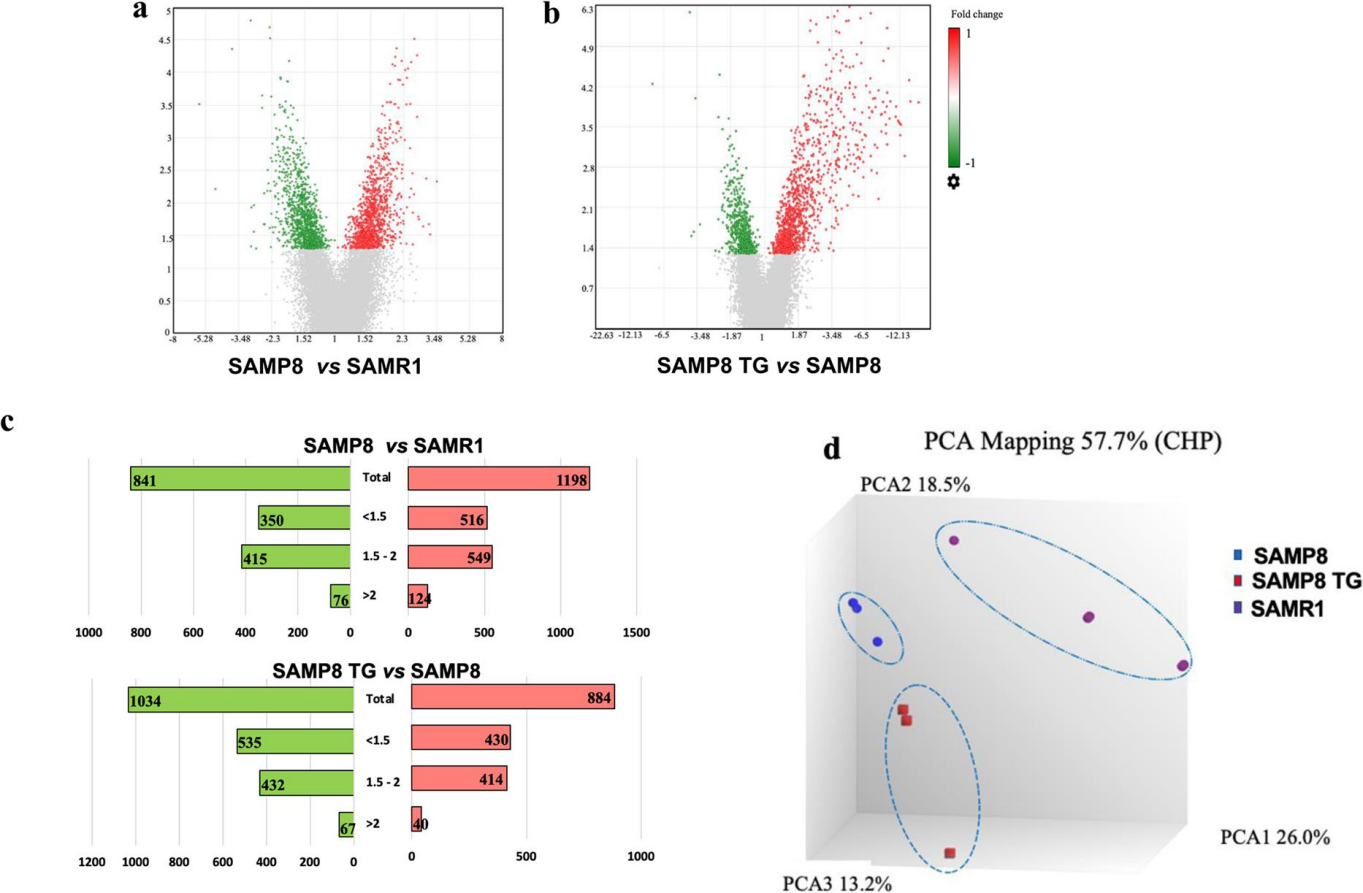
Microarray gene expression profile of TG treatment in SAMP8 mice. The volcano plot displays DEGs between **(a)** the SAMP8 nontreated group *vs.* SAMR1 group and **(b)** the SAMP8 (5 mg/kg) TG-treated group *vs.* SAMP8 nontreated group. A volcano plot was generated using Transcriptome analysis console version 4 software. The vertical axis (y-axis) corresponds to –log10 (*P* value), whereas the horizontal axis (x-axis) displays linear fold change. The red and green dots represent the upregulated and downregulated genes, respectively. **c** The bar graph shows the numbers of significantly up and downregulated DEGs and the distribution of fold changes (> 2, 1.5 ~ 2, < 1.5) of DEGs in the different comparison conditions. The red and green bars represent the up and downregulated DEGs, respectively. **d** Cluster plot based on Principal Component Analysis (PCA) showing uncorrelated variables between the control and treated groups. All the groups (SAMP8 and TG) showed different gene expression compared to the SAMR1 group.

Using the Clariom S Assay microarray tool, we obtained transcriptome-wide gene-level expression data from a total of 22,260 well-annotated genes. Compared to the SAMR1 group, we identified 2039 unique DEGs in the SAMP8 group, with 1198 upregulated and 841 downregulated DEGs, as shown in the volcano plot (Fig. 2a). Similarly, when comparing the TG-treated SAMP8 group to the SAMP8 group, we observed 1918 unique DEGs with 884 upregulated and 1034 downregulated (Fig. 2b).

The butterfly chart in Fig. 2c shows the distribution of fold changes of the DEGs. Compared to the SAMR1 group, the SAMP8 group had 124 upregulated and 76 downregulated DEGs with FC > 2. Likewise, the TG-treated SAMP8 group had 40 upregulated and 67 downregulated DEGs with FC > 2 compared to the nontreated SAMP8 group.

Figure 2d presents a principal component analysis (PCA) showcasing the first three components plotted based on the signal (CHP) data probe sets, which capture the highest variance observed among the groups. Each group is represented by a distinct color on the PCA graph, with SAMR1 in purple, SAMP8 in blue, and TG-treated SAMP8 in red. Interestingly, we observed that all three groups displayed linear uncorrelation, providing further evidence that TG treatment modulated the gene expression profile in SAMP8 mice. Top 10 up and downregulated DEGs in TG-treated SAMP8 mice (*vs*. nontreated SAMP8) and their biological functions are presented in Tables 1 and 2, respectively.

**Table 1:** Top 10 upregulated DEGs in TG-treated SAMP8 mice (*vs.* nontreated SAMP8) and their biological functions

Gene symbol	Description	Fold change	P-value	Biological process (BP)
*Dbi*	Diazepam binding inhibitor	2.5	0.0003	Learning and memory, glial cell proliferation, regulation of synaptic transmission, GABAergic synapse, skin development, positive regulation of lipid biosynthetic process, long-term synaptic potentiation, positive regulation of CoA-transferase activity, positive regulation of phospholipid transport
*Fabp2*	Fatty acid binding protein 2, intestinal	2.48	0.0081	Fatty acid metabolic process, long-chain fatty acid transport, intestinal lipid absorption
*Il2ra*	Interleukin 2 receptor, alpha chain	2.39	0.0117	Inflammatory response to an antigenic stimulus, regulation of T cell tolerance induction, inflammatory response, positive regulation of T cell proliferation, lymphocyte proliferation, negative regulation of inflammatory response
*Atp5l*	ATP synthase, H + transporting, mitochondrial F0 complex, subunit G	2.34	9.14E-05	ATP biosynthetic process, ATP metabolic process
*Mrpl20*	Mitochondrial ribosomal protein L20	2.23	0.0035	Ribosomal large subunit assembly, translation, ribosomal large subunit assembly, translation, mitochondrial translation
*Gpx4*	Glutathione peroxidase 4	2.07	0.0003	Lipid metabolic process, glutathione metabolic process, response to oxidative stress
*Sytl2*	Synaptotagmin-like 2	2.04	0.0335	Intracellular protein transport, exocytosis, vesicle docking involved in exocytosis, positive regulation of mucus secretion, protein localization to the plasma membrane
*Mrpl12*	Mitochondrial ribosomal protein L12	2.02	0.0183	Mitochondrial transcription, translation, translation, mitochondrial translation, positive regulation of DNA-templated transcription,
*Tnfrsf23*	Tumor necrosis factor receptor superfamily, member 23	1.93	0.0003	Regulation of stress-activated MAPK cascade, negative regulation of the apoptotic process, motor neuron apoptotic process, extrinsic apoptotic signaling pathway in absence of ligand
*Igfbp6*	Insulin-like growth factor binding protein 6	1.93	0.0077	Positive regulation of MAPK cascade, regulation of insulin-like growth factor receptor signaling pathway

Statistically significant value was defined by *p* < 0.05. The biological process terms were obtained by utilizing DAVID online tool http://david.ncifcrf.govt/

**Table 2:** Top 10 downregulated DEGs in TG-treated SAMP8 mice (*vs.* nontreated SAMP8) and their biological functions

Gene symbol	Description	Fold change	*P*-value	Biological process (BP)
*Ptgds*	Prostaglandin D2 synthase (brain)	-5.72	0.0003	Prostaglandin biosynthetic process, mast cell degranulation, prostaglandin metabolic process
*Acta2*	Actin, alpha 2, smooth muscle, aorta	-4.66	0.0062	Muscle contraction, vascular associated, smooth muscle contraction
*Igf2*	Insulin-like growth factor 2	-3.78	4.39E-05	Insulin-like growth factor receptor signaling pathway, negative regulation of natural killer cell-mediated cytotoxicity, response to nicotine, insulin receptor signaling pathway via phosphatidylinositol 3-kinase
*Sema5a*	Transmembrane domain (TM) and short cytoplasmic domain	-2.97	0.0117	Negative regulation of axon extension involved in axon guidance, regulation of actin filament depolymerization
*Prkcd*	Protein kinase C, delta	-2.88	0.0109	IL10 production, IL12 production, positive regulation of superoxide anion generation
*Arel1*	Apoptosis resistant E3 ubiquitin protein ligase 1	-2.59	0.0004	Proteasome-mediated ubiquitin-dependent protein catabolic process, regulation of inflammatory response, protein K11-linked ubiquitination, protein K33-linked ubiquitination
*Glb1l*	Galactosidase, beta 1-like	-2.43	0.0066	Carbohydrate metabolic process
*Actn4*	Actinin alpha 4	-2.41	0.0031	Tumor necrosis factor-mediated signaling pathway, positive regulation of NIK/NFκB signaling
*Tfcp2*	Transcription factor ceruloplasmin 2	-2.38	0.005	Copper ion transport, iron ion transport
*Dcc*	Deleted in colorectal carcinoma	-2.35	2.04E-05	Dorsal/ventral axon guidance, anterior/posterior axon guidance positive regulation of ERK1 and ERK2 cascade, cell–cell adhesion, postsynaptic modulation of chemical synaptic transmission

Statistically significant value was defined by *p* < 0.05. The biological process terms were obtained by utilizing DAVID online tool http://david.ncifcrf.govt/

### TG altered accelerated aging-associated gene expression in SAMP8 mice

**Fig. 3:**
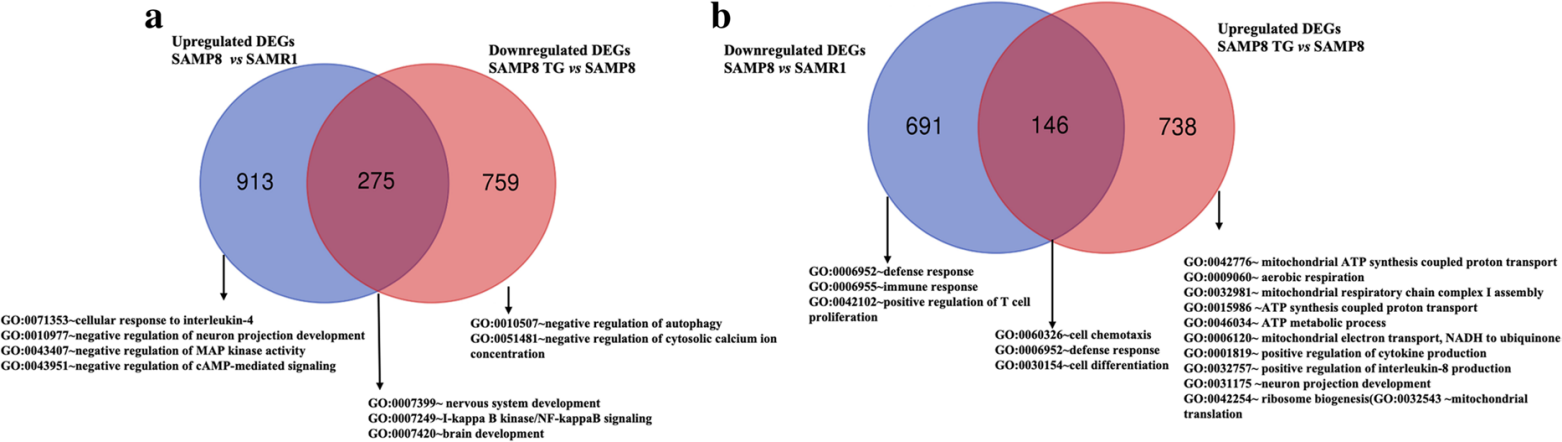
Venn diagram representing the unique and common gene sets. **a** The blue circle represents upregulated DEGs in the SAMP8 compared to the SAMR1 The red circle represents downregulated DEGs in SAMP8 TG-treated group compared to the SAMP8 nontreated group. **b** The blue circle denotes downregulated DEGs in SAMP8 compared to SAMR1. The red circle represents upregulated DEGs in SAMP8 TG-treated group compared to the SAMP8.

Next, we examined the biological events associated with accelerated aging in SAMP8 mice that were significantly affected by TG treatment. We constructed a Venn diagram to identify the gene sets that were shared between the SAMP8 vs. SAMR1 and TG-treated SAMP8 vs. SAMP8 comparison conditions while exhibiting expression in the opposite direction. We then conducted an overrepresentation analysis to identify gene ontology biological processes (GOBPs) that were significantly enriched by both the shared and unique gene sets (Fig. 3a, b).

Figure 3a shows that (GO:0071353; *n* = 5) cellular response to interleukin-4, (GO:0010977; *n* = 9) negative regulation of neuron projection development, (GO:0043407; *n* = 7) negative regulation of MAP kinase activity, (GO:0043951; *n* = 4) negative regulation of cAMP-mediated signaling were among the most significantly affected GOBP terms by the unique set of upregulated DEGs in the SAMP8 group compared to SAMR1 (*n* = 931). TG treatment significantly downregulated 275 genes that were initially upregulated in the nontreated SAMP8 group compared to SAMR1. The common set of genes (*n* = 275) exhibited significant enrichment (*P* < 0.05) in BP terms such as (GO:0007399; *n* = 11) nervous system development, (GO:0007249; *n* = 4) I-кB kinase/NFкB signaling, and (GO:0007420; *n* = 9) brain development. Additionally, the unique downregulated gene sets (*n* = 759) in the TG-treated SAMP8 group significantly enriched BP terms associated with (GO:0010507; *n* = 6) negative regulation of autophagy an (GO:0051481; *n* = 5) negative regulation of cytosolic calcium ion concentration.

On the other hand, enriched GOBP terms by the unique set of downregulated DEGs in SAMP8 mice (*n* = 691) included (GO:0006952; *n* = 10) defense response, (GO:0006955; *n* = 23) immune response, and (GO:0042102; *n* = 8) positive regulation of T cell proliferation. TG treatment led to the significant downregulation of genes (*n* = 146) that were initially upregulated in SAMP8 mice compared to SAMR1, among which (GO:0060326; *n* = 4) cell chemotaxis, (GO:0006952; *n* = 8) defense response, and (GO:0030154; *n* = 11) cell differentiation were most significant.

Additionally, the significantly upregulated unique DEGs in the TG-treated SAMP8 group (*n* = 738) enriched BP terms of  (GO:0042776; *n* = 26) mitochondrial ATP synthesis coupled proton transport, (GO:0009060; *n* = 20) aerobic respiration, (GO:0032981; *n* = 18) mitochondrial respiratory chain complex I assembly, (GO:0015986; *n* = 8) ATP synthesis coupled proton transport, (GO:0046034; *n* = 11) ATP metabolic process, (GO:0006120; *n* = 8) mitochondrial electron transport, NADH to ubiquinone, (GO:0001819; *n* = 7) positive regulation of cytokine production, (GO:0032757; *n* = 6) positive regulation of interleukin-8 production, (GO:0031175; *n* = 12) neuron projection development, (GO:0042254; *n* = 9) ribosome biogenesis, and (GO:0032543; *n* = 9) mitochondrial translation.

### TG regulated a wide range of biological processes in the hippocampus of SAMP8 mice

**Fig. 4:**
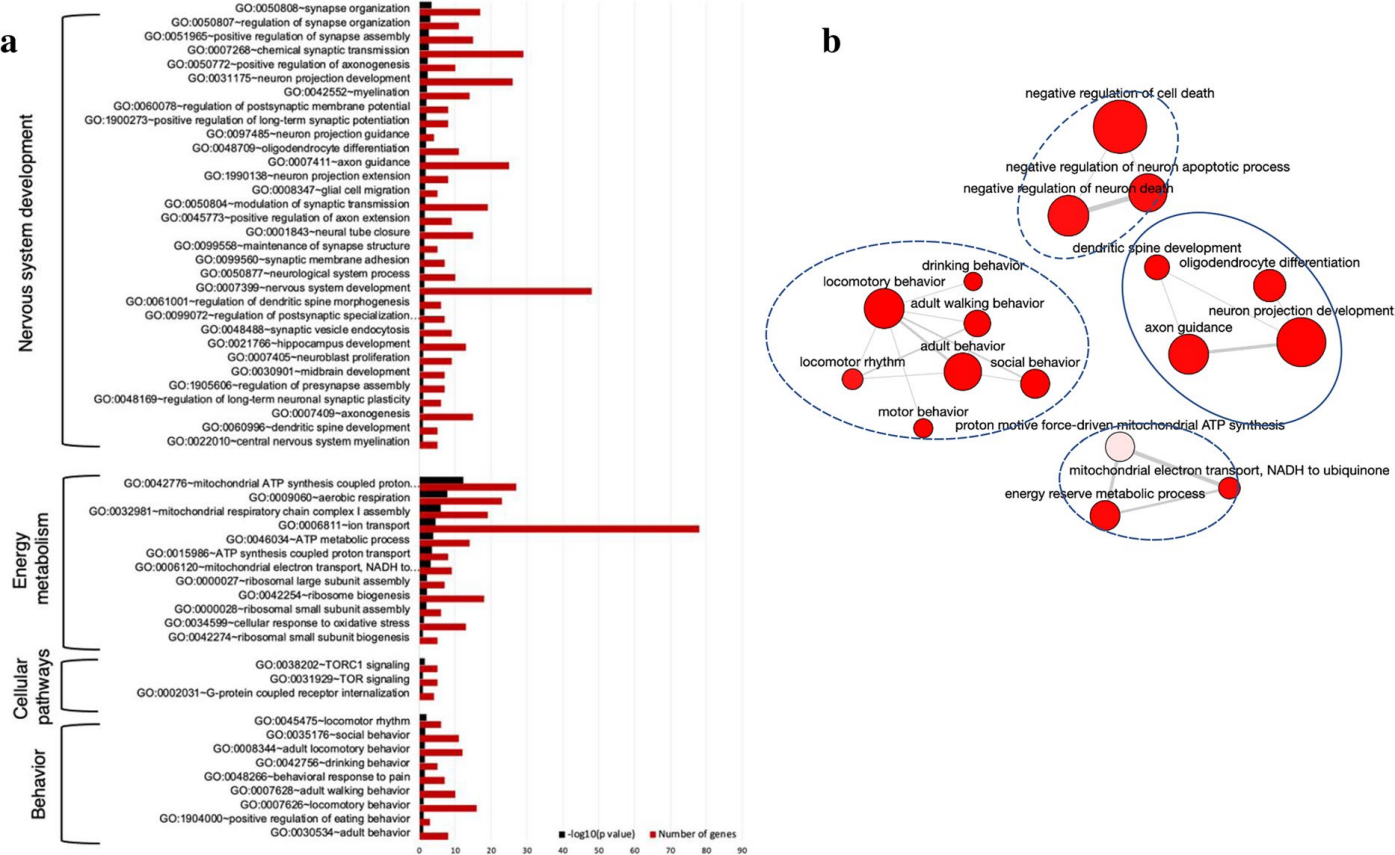
Effects of TG on the biological process of SAMP8 mice. **a** Bar graph showing the top significantly enriched biological process gene ontology (GOBP) terms by the DEGs. GOBP terms were identified using DAVID online software. **b** An interactive graph showing the interrelation of the significantly enriched GOBP terms. The bubble color indicates the *P* value; the bubble size indicates the frequency of the GO term. Highly similar GO terms are linked by edges in the graph, where the line width indicates the degree of similarity

Next, we explored the comprehensive range of bioactivities exerted by TG in SAMP8 mice. All DEGs in the TG-treated group compared to nontreated SAMP8 were subjected to GO analysis. The enriched GOBP terms (*P* < 0.05), along with their corresponding negative log-transformed significance values (-log10 P value) and the numbers of up and downregulated DEGs associated with each term, are presented in Fig. 4a and Supplementary file 1.

All significant DEGs of TG-treated SAMP8 *vs.* SAMP8 were applied to analyze biological processes divided into four distinct clusters, involved in behavior, nervous system development, energy metabolism, and signaling pathways.

Top significantly enriched biological processes related to nervous system development are the (GO:0050808; *n* = 17) synapse organization, (GO:0050807; *n* = 11) regulation of synapse organization, (GO:0051965; *n* = 15) positive regulation of synapse assembly, (GO:0007268; *n* = 29) chemical synaptic transmission, (GO:0050772; *n* = 10) positive regulation of axonogenesis, (GO:0031175; *n* = 26) neuron projection development, (GO:0042552; *n* = 14) myelination, (GO:1900273; *n* = 8) positive regulation of long-term synaptic potentiation, (GO:0060078; *n* = 8) regulation of postsynaptic membrane potential, (GO:0097485; *n* = 4) neuron projection guidance, (GO:0048709; *n* = 11) oligodendrocyte differentiation, (GO:0007411; *n* = 25) axon guidance, (GO:1990138; *n* = 8) neuron projection extension, (GO:0008347; *n* = 5) glial cell migration, (GO:0050804; *n* = 19) modulation of synaptic transmission.

Other enriched BR terms included (GO:0045773; *n* = 9) positive regulation of axon extension, (GO:0001843; *n* = 15) neural tube closure, (GO:0099558; *n* = 5) maintenance of synapse structure, (GO:0099560; *n* = 7) synaptic membrane adhesion, (GO:0050877; *n* = 10) neurological system process, (GO:0007399; *n* = 48) nervous system development, (GO:0061001; *n* = 6) regulation of dendritic spine morphogenesis, (GO:0099072; *n* = 7) regulation of postsynaptic specialization membrane neurotransmitter receptor levels, (GO:0048488; *n* = 9) synaptic vesicle endocytosis, (GO:0021766; *n* = 13) hippocampus development, (GO:0007405; *n* = 9) neuroblast proliferation, (GO:1905606; *n* = 7) regulation of presynapse assembly, (GO:0030901; *n* = 7) midbrain development, (GO:0048169; *n* = 6) regulation of long-term neuronal synaptic plasticity, (GO:0007409; *n* = 15) axonogenesis, (GO:0022010; *n* = 5) central nervous system myelination, and (GO:0060996; *n* = 5) dendritic spine development.

Energy metabolism-related pathways were (GO:0042776; *n* = 27) mitochondrial ATP synthesis coupled proton transport, (GO:0009060; *n* = 23) aerobic respiration, (GO:0032981; *n* = 19) mitochondrial respiratory chain complex I assembly, (GO:0006811; *n* = 78) ion transport, (GO:0046034; *n* = 14) ATP metabolic process, (GO:0015986; *n* = 8) ATP synthesis coupled proton transport, (GO:0006120; *n* = 9) mitochondrial electron transport, NADH to ubiquinone, (GO:0000027; *n* = 7) ribosomal large subunit assembly, (GO:0042254; *n* = 18) ribosome biogenesis, (GO:0000028; *n* = 6) ribosomal small subunit assembly, (GO:0034599; *n* = 13) cellular response to oxidative stress, and (GO:0042274; *n* = 5) ribosomal small subunit biogenesis. Important signaling pathway-related biological processes were (GO:0038202; *n* = 5) TORC1 signaling (GO:0031929; *n* = 5) TOR signaling, and (GO:0002031; n = 4) G-protein coupled receptor internalization.

 Behavior-related pathways were (GO:0045475; *n* = 6) locomotor rhythm, (GO:0035176; *n* = 11) social behavior, (GO:0008344; *n* = 12) adult locomotory behavior, (GO:0042756; *n* = 5) drinking behavior, (GO:0048266; *n* = 7) behavioral response to pain, (GO:0007628; *n* = 10) adult walking behavior, (GO:0007626; *n* = 16) locomotory behavior, (GO:1,904,000; *n* = 3) positive regulation of eating behavior, and (GO:0030534; *n* = 8) adult behavior.

A network plot of GOBP terms was generated to visualize the interactive relationships among the most significantly enriched BP terms affected by TG treatment (Fig. 4b). The plot highlights the correlation between these enriched GO terms forming functional clusters, encompassing diverse aspects, including nervous system development, behavior, signaling pathways related to cell death/apoptosis inhibition, and energy metabolism.

### Dimensionality reduction approach revealed a prominent anti-inflammatory property of TG in the hippocampus of SAMP8 mice 

**Fig. 5:**
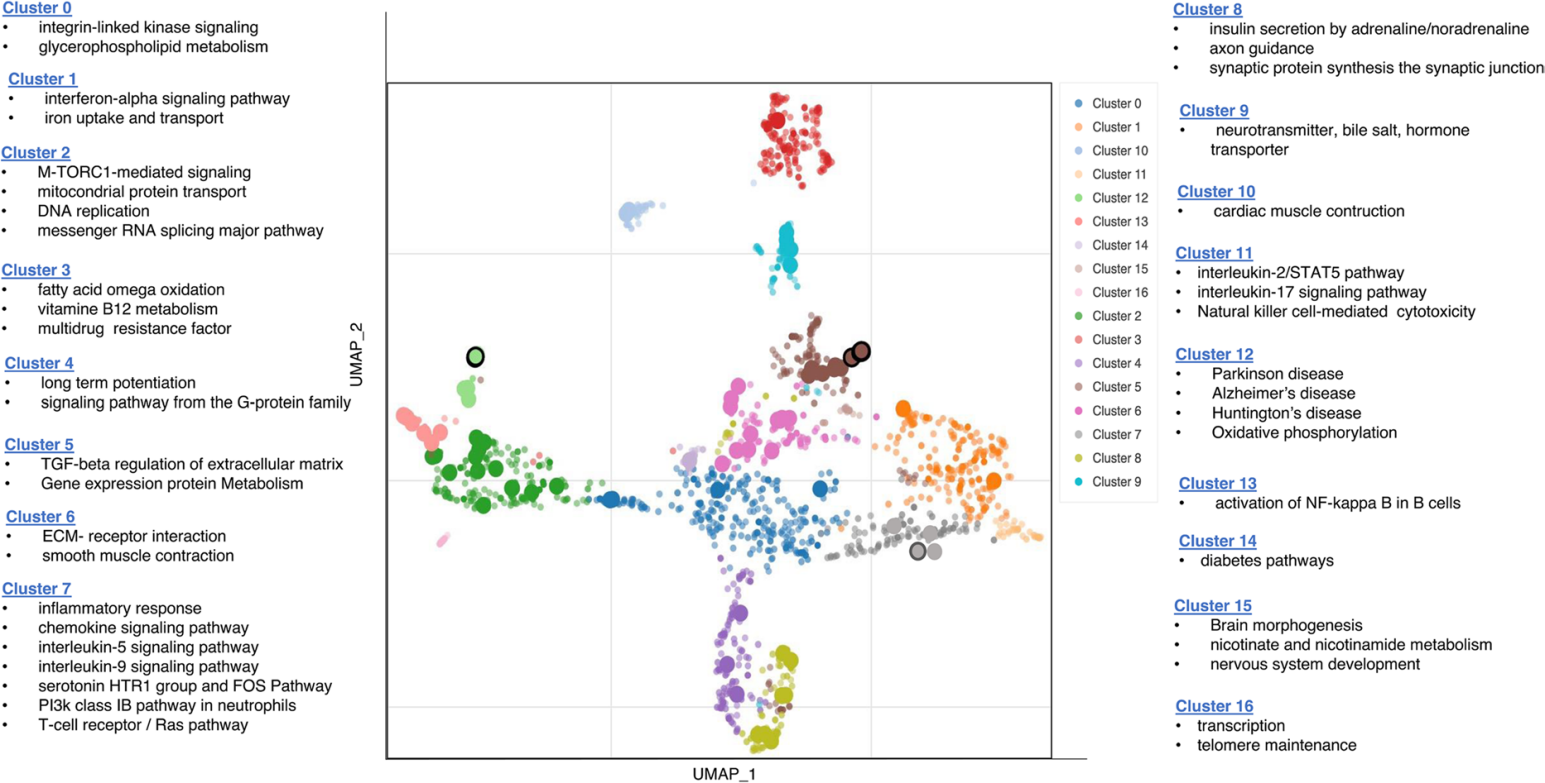
Significantly enriched GO terms in the BioPlanet_2019 gene set library. Each color of the scatter plot represents a term in the library. Term frequency-inverse document frequency (TF-IDF) values were computed for the gene set corresponding to each term, and UMAP was applied to the resulting values. The terms are plotted based on the first two UMAP dimensions. Terms are colored by automatically identified clusters computed with the Leiden algorithm applied to the TF-IDF values. The darker and more extensive the point, the more significantly enriched the term

To identify functionally related pathways in the TG-treated group (vs. nontreated SAMP8 group), we applied the UMAP dimensionality reduction technique. By plotting the Bioplanet 2019 gene set library terms on the first two UMAP dimensions, we were able to identify the most prominent clusters and pathway terms (Fig. 5).

We summarized 16 significantly enriched pathway clusters that share similar gene sets. Among these clusters, inflammatory pathways were predominant in clusters 1, 5, 7, 11, and 13, representing interferon alpha, TGFβ, ILs 2, 5, 9, and 17, and NFкB pathways. Clusters 4, 8, and 9 were associated with synaptic function and neurotransmitter-related pathways. Additionally, cluster 15 highlighted pathways related to brain development.

(Data presented in Supplementary excel file).

### TG regulated the expression of inflammation-associated genes in the hippocampus of SAMP8 mice

**Fig. 6:**
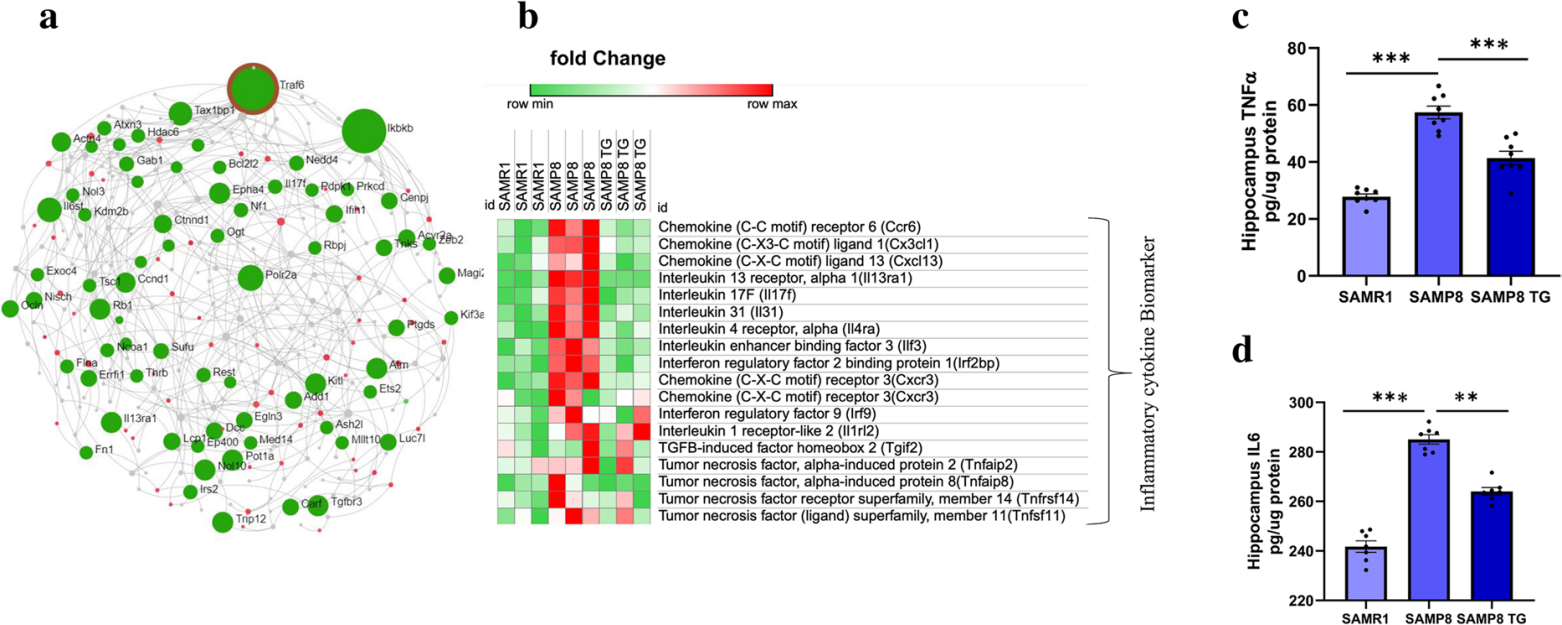
TG suppresses inflammatory cytokines in the mice hippocampus. **a** Generic first-order Protein–Protein Interaction (PPI) network by the DEGs between SAMP8 TG-treated group *vs.* SAMP8 nontreated group. An interaction network was generated for the molecular function of cytokine-related biomarkers. cytokine binding and cytokine receptor binding. Downregulated DEGs are shown in green, and upregulated DEGs are presented in red. The size of the node denotes the number of degrees. PPI was generated by using online software. **b** Heatmap was generated from microarray data showing the relative expression intensity of genes involved in neuroinflammation; Heatmap was generated by using the online visualization software Morpheus (**c)** ELISA measured TNFα, (**d**) IL6 in brain tissues. Values are presented as mean ± *SEM* (n = 6 ~ 8 animals per group). Statistical analysis was performed by using one-way ANOVA followed by Fisher’s LSD test: *P < 0.05; **P < 0.01; ***P < 0.001

We generated a PPI interaction network to identify the hub genes associated with the biological functions of TG. First, we built the first-order undirected network consisting of all DEGs (Supplementary Fig. S5). TNFα, Receptor Associated Factor 6 (*Traf6*) was the top hub node with the highest degree (degree = 87, betweenness = 221,575.4). All hub nodes are shown in the Supplementary Fig. S5. As inflammatory pathways were the key functional clusters observed in the UMAP dimension reduction model, we limited the PPI network to molecular functions of cytokine binding (hits = 225, adjusted *P* value = 0.00144) and cytokine receptor binding (hits = 53, adjusted *P* value = 0.000032). We again found that *Traf6* is the top hub gene with the highest degree (degree = 29, betweenness = 6443.59, Fig. 6a). Inhibitor of NF-κB Kinase Subunit Beta (*Ikbkb)* was another top hub gene (degree = 12, betweenness = 1763.95).

The heatmap (Fig. 6b**)** shows the relative intensity of the genes that regulated in the opposite direction, both in SAMR1 and TG-treated SAMP8, compared to SAMP8. Presented genes are mainly involved in neuroinflammation, including chemokine (C–C motif) receptor 6 (*Ccr6*); chemokine (C-X3-C motif) ligand 1(*Cx3cl1*); chemokine (C-X-C motif) ligand 13 (*Cxcl13*); IL13 receptor, alpha 1(*Il13ra1*); IL17F (*Il17f)*; IL31 (*Il31*); IL4 receptor, alpha (*Il4ra*); IL enhancer-binding factor 3 (*Ilf3*); interferon regulatory factor 2 binding protein 1 (*Irf2bp*); chemokine (C-X-C motif) receptor 3 (*Cxcr3*); interferon regulatory factor 9 (*Irf9*); IL12 receptor, beta 2 (*Il12rb2*); IL 1 receptor-like 2 (*Il1rl2*); TGFβ-induced factor homeobox 2 *(Tgif2*); TNF, alpha-induced protein 2 (*Tnfaip2*); TNF, alpha-induced protein 8 (*Tnfaip8)*; TNF receptor superfamily, member 14 (*Tnfrsf14*) and TNF (ligand) superfamily, member 11 (*Tnfsf11*). We observed that proinflammatory cytokine-related genes were downregulated in TG-treated SAMP8 mice. This result indicates the anti-inflammatory properties of TG (Heatmap data presented in Supplementary excel file).

To validate the anti-inflammatory properties of TG in SAMP8 mice, we conducted ELISA to quantify the protein levels of pro-inflammatory cytokines- TNFα and IL6 (Fig. 6c, d). We observed a significant increase in the levels of TNFα (Δmean = 29.59; *P* < 0.001) and IL6 (Δmean = 45.70; *P* < 0.001) in the hippocampus of nontreated SAMP8 mice compared to SAMR1 mice. However, TG treatment resulted in a significant reduction in both TNFα (Δmean = 16.07; *P* < 0.001) and IL6 levels (Δmean = 20.58; *P* < 0.01) in SAMP8 mice when compared to the nontreated SAMP8 group (All values are presented in the Supplementary Table 1).

Neuroinflammation in the aging brain has been reported to exhibit similar biological responses triggered by bacterial lipopolysaccharides (LPS). Additionally, it has been observed that LPS-induced inflammation disrupts long-term potentiation and cognitive function in the hippocampus. Thus, in order to further confirm the ability of TG to alleviate neuroinflammation, we examined the effect of TG on the expression of TNFα mRNA in LPS-induced human neuro typic SH-SY5Y cells. We found that TG treatment significantly reduced the expression level of TNFα by approximately 72 ± 2.3% within a 24-h treatment period, suggesting TG treatment effectively reduces inflammation induced by LPS in SH-SY5Y cells (Fig. S2).

### TG modulated synapse-related functions in the hippocampus of SAMP8 mice 

**Fig. 7:**
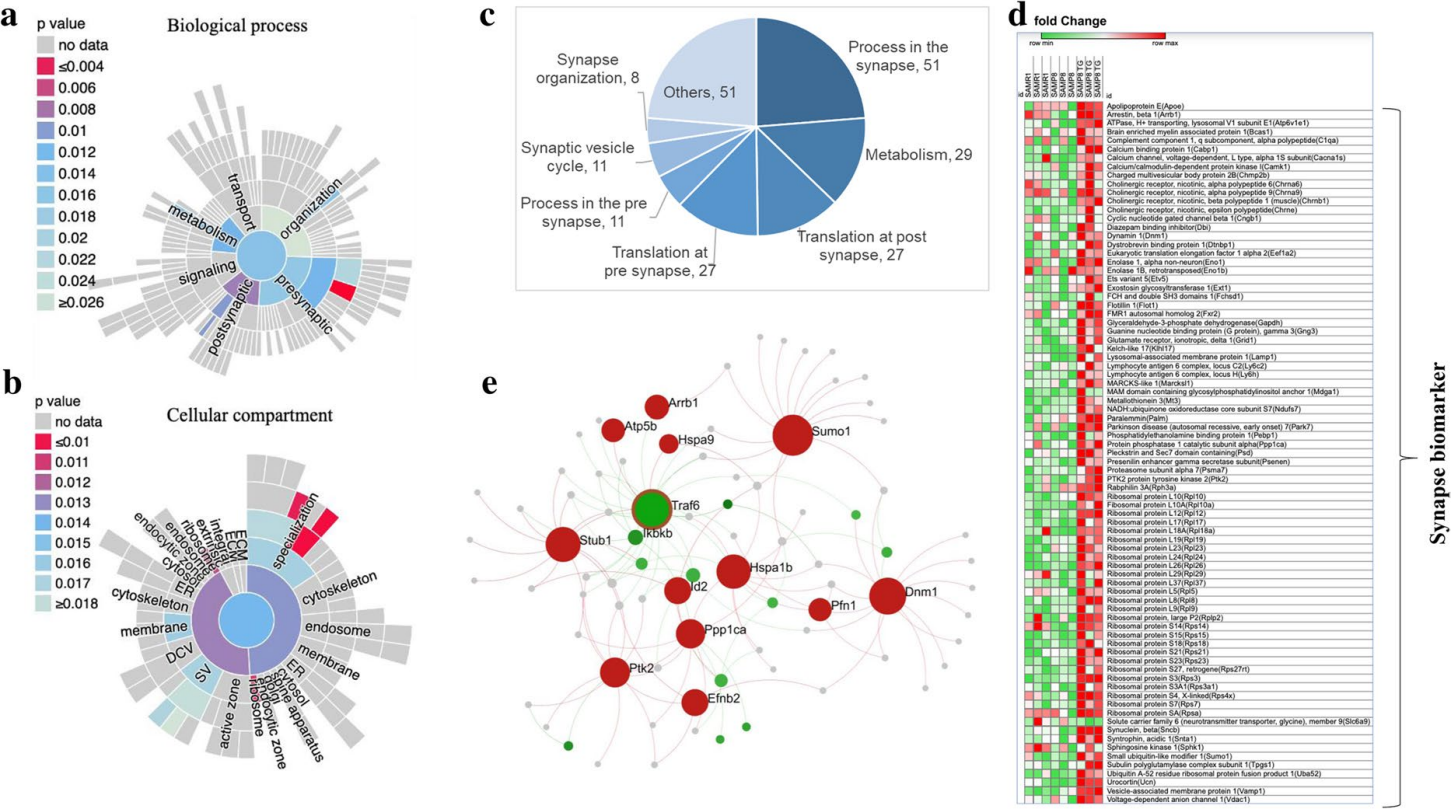
TG facilitates synapse-related genes in the hippocampus. **a** Sunburst plot showing significantly enriched biological process (BP) terms based on the synapse-specific SynGO database annotation **(b)** Sunburst plot showing enriched cellular component (CC) terms based on the synapse-specific SynGO database annotation. The color encodes the significance of the enriched *P* value. **c** pie chart showing the number of genes related to synapse-specific BP terms. **d** Heatmap was generated by analyzing microarray data showing the relative expression intensity of genes involved in neurotransmitter release. **e** The PPI network of the *Traf6* gene shows its interaction with several synapse-related upregulated genes

Given the cluster analysis results indicating a potential effect of TG on synaptic regulation (as shown in Fig. 5), we conducted a further analysis using SynGO, an evidence-based and expert-curated resource that focuses on synaptic locations and functions. We visualized the significantly overrepresented (*P* < 0.05) synaptic functions and subcellular locations using sunburst plots (Fig. 7a, b).

The increased number of significant synapse-specific biological process (BP) terms was the process in the synapse (*P* < 0.02; n = 51), metabolism (*P* < 0.01; *n* = 29). Additionally, the top number of significant cellular component (CC) terms included synapse (*P* < 0.014; n = 77) post synapse *P* < 0.013; n = 58).

The pie chart represents the number of DEGs involved in maintaining different synapse-specific BPs (Fig. 7c); among them, 51 genes were involved in the process in the synapse, 29 in metabolism, 27 in the translation of post-synapse and the translation of pre-synapse each.

Next, we examined the gene expression profiling of synapse-related genes, specifically focusing on genes involved in the signaling, metabolism, organization, and transport of synapses. The heatmap in Fig. 7d displays the DEGs that were downregulated in SAMP8 and SAMR1 mice but upregulated in TG-treated SAMP8 mice. Several cholinergic receptors were significantly upregulated by TG treatment, such as a cholinergic receptor, nicotinic, alpha polypeptide (*Chrna)* 6 (*Chrna6*), *Chrna9*, cholinergic receptor, nicotinic, beta polypeptide 1 (muscle) (*Chrnb1*) and cholinergic receptor, nicotinic, epsilon polypeptide (*Chrne*).

Next, we constructed a PPI network targeted for the cellular components of the synapse (hits = 134, adjusted p-value = 1.03e-11), synapse part (hits = 92, adjusted p-value = 1.09e-9), and synaptic vesicle (hits = 20, adjusted p-value = 0.03) (Fig. 7e). We identified that *Traf6* is one of the top hub genes, which showed interaction with several synapse-related upregulated genes, such as Dynamin 1 **(***Dnm1)***,** STIP1 Homology, and U-Box Containing Protein 1 (*Stub1*), and Small Ubiquitin Like Modifier 1 (*Sumo1*).

### TG upregulated neurotransmitter levels in SAMP8 mice hippocampus

Since pathway clustering and synapse-specific functional enrichment analysis predicted that TG administration might improve synaptic function in SAMP8 mice, we conducted further validation by examining the effect of TG on neurotransmitter levels.

**Fig. 8:**
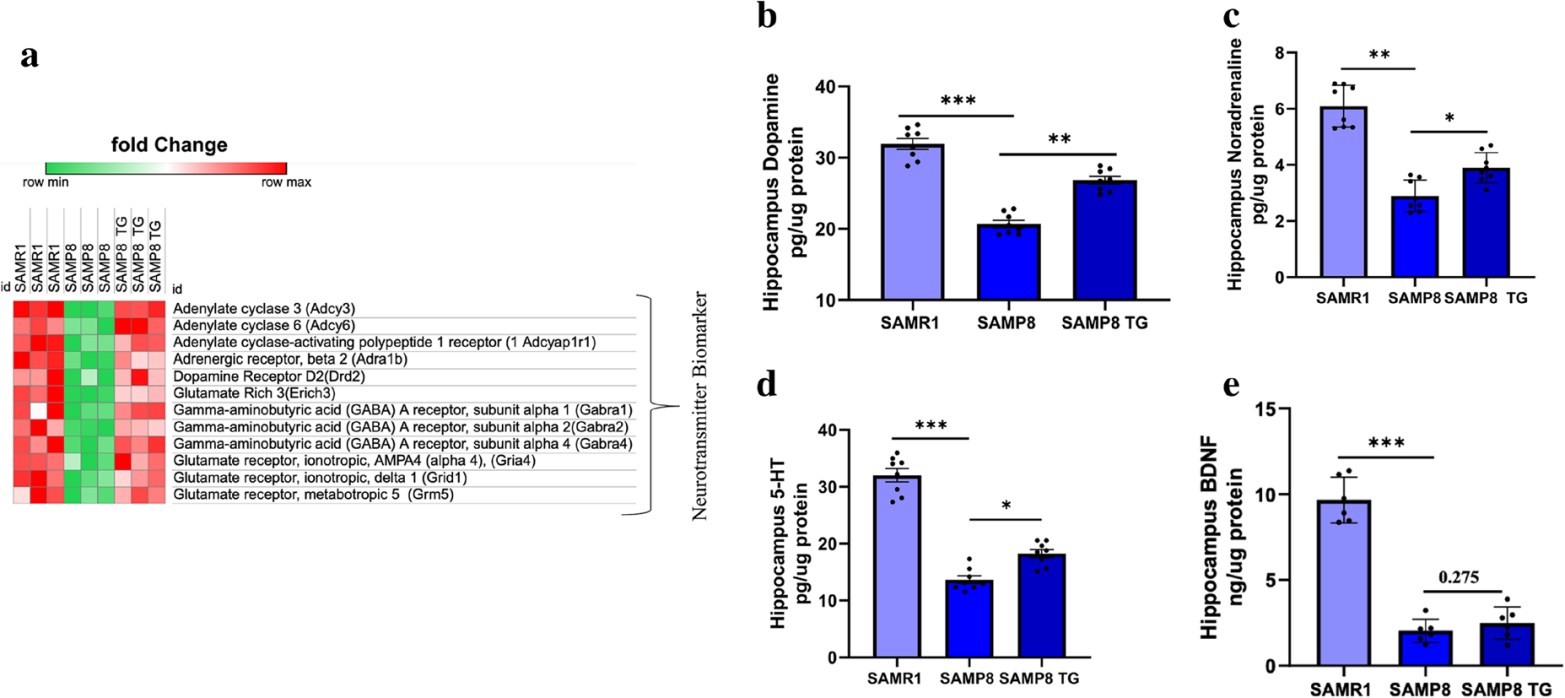
TG elevates neurotransmitter levels in the hippocampus. **a** Heatmap showing significantly enriched DEGs associated with neurotransmitter releases in the hippocampus. **b** DA, **(c)** NA, **(d)** 5-HT, and (**e)** BDNF in brain tissues were measured by ELISA. Values are presented as mean ± *SEM* (n = 6 ~ 8 animals per group). Comparisons were performed using one-way ANOVA followed by Fisher’s LSD test: *P < 0.05; **P < 0.01; ***P < 0.001.; ns, non-significant

First, we assessed the relative expression levels of genes associated with neurotransmitter release that exhibited similar direction regulation in TG-treated SAMP8 and SAMR1 mice compared to nontreated SAMP8 mice (Fig. 8a). The heatmap displays the top significantly upregulated DEGs, including Adenylate Cyclase 6 (*Adcy6*), Adenylate Cyclase-Activating Polypeptide 1 Receptor 1 (*Adcyap1r1*), Adrenergic Receptor, Alpha 1D (*Adra1d*), Adrenergic Receptor, Beta 2 (*Adrb2*), Gamma-Aminobutyric Acid (GABA) A Receptor, Subunit Alpha 1 (*Gabra1*), GABA A Receptor, Subunit Alpha 2 (*Gabra2*), GABA A Receptor, Subunit Alpha 4 (*Gabra4*), Glutamate Receptor, Ionotropic, AMPA4 (Alpha 4) (*Gria4*), Glutamate Receptor, Ionotropic, Delta 2 (*Grid2*), and Glutamate Receptor, Metabotropic 5 (*Grm5*). Further details can be found in the Supplementary file.

Subsequently, we validated the observed potential of TG in neurotransmitter regulation by performing an ELISA test to measure the levels of neurotransmitters such as DA, NE, and 5-HT in the total protein extracted from the hippocampus of mice. All three neurotransmitters were significantly decreased in nontreated SAMP8 mice compared to SAMR1 mice (Δmean = 11.03; *P* < 0.001; Δmean = 3.07; *P* < 0.001; and Δmean = 18.37; *P* < 0.001 for DA, NE, and 5-HT levels, respectively). However, TG treatment significantly elevated the levels of all three neurotransmitters compared to the nontreated SAMP8 group (Δmean = 4.91, *P* < 0.01; Δmean = 1.00, *P* < 0.05; and Δmean = 4.5, *P* < 0.05 for DA, NE, and 5-HT levels, respectively) (Fig. 8b, c, and d).

Additionally, we examined the effect of TG treatment on the level of the neurotrophic factor BDNF in mice hippocampus. Similar to the neurotransmitter levels, the BDNF level in the SAMP8 group was significantly lower compared to SAMR1 mice (Δmean = 7.87; *P* < 0.001). TG treatment could slightly increase the BDNF level in SAMP8 mice compared to the nontreated group (Δmean = 0.69, *P* = 0.27); however, this change did not reach statistical significance (Fig. 8e). (Detailed values of the ELISA tests can be found in Supplementary Table 1).

## Discussion

**Fig. 9:**
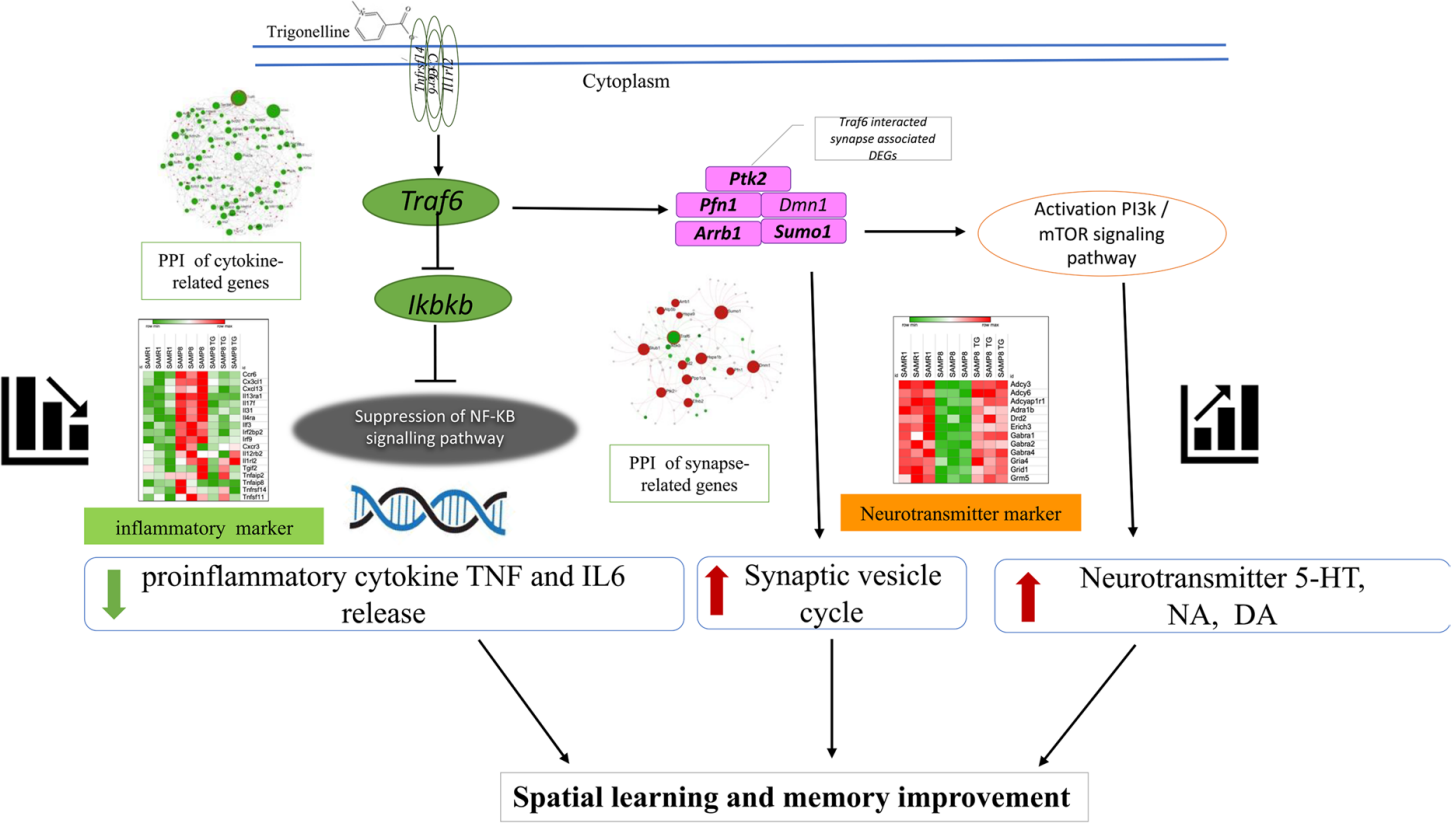
Schematic diagram representing the mechanism of action of TG

In this study, we have demonstrated that oral administration of TG over 30 days significantly improved the memory acquisition of 16-week-old SAMP8 mice, as observed in the hippocampal-dependent memory task MWM test, indicating its potential in alleviating cognitive decline associated with accelerated aging. Furthermore, our integrated analysis of the whole-genome transcriptome and protein levels in the mice hippocampus revealed that TG administration suppressed neuroinflammation and enhanced neurotransmitter release, contributing to the observed improvements in cognitive function. Moreover, our untargeted analysis of the transcriptome data provided insights into the potential mechanism by which TG exerts its effects. We identified the Traf6-mediated signaling pathway of NF-κB as a potential target of TG, leading to reduced synthesis of proinflammatory cytokines like TNFα and IL6. Moreover, TG appeared to modulate the interplay between Traf6 and various neurotransmitters, facilitating their release and ultimately enhancing learning and memory formation in SAMP8 mice. Overall, our study provides valuable insights into the therapeutic potential of TG in ameliorating cognitive decline associated with accelerated aging, highlighting its ability to target neuroinflammation, synaptic function, and neurotransmitter release in the hippocampus (Fig. 9).

### TG-induced reduction of neuroinflammation in the hippocampus

Neuroinflammation is a vital risk factor for developing cognitive aging and neurodegenerative disease. Inflammatory cytokines are considered a hallmark of neuronal cell death and dysfunction [19, 33]. The role of the inflammatory response includes proinflammatory cytokines (IL1b, IL2, IL6, and TNFα), chemokines (CCL2, CCL5, and CXCL1), secondary messengers (NO and prostaglandins), and reactive oxygen species (ROS) most of these mediator factors are produced by activated resident cells of the central nervous system [34]. We found that TG treatment significantly reduces TNFα and IL6 protein levels in SAMP8 mice hippocampus (Fig. 6c, d). Additionally, it also suppressed LPS-induced TNFα mRNA expression in SH-SY5Y cells (Fig. S2), suggesting the beneficial effect of TG in suppressing neuroinflammation [33].

To elucidate the underlying molecular mechanism, we conducted a comprehensive analysis of whole-genome data from the hippocampi of TG-treated and nontreated SAMP8 mice. Through functional cluster analysis using the UMAP dimension reduction approach, we identified that the primary bioactivity of TG may be its anti-inflammatory function as numerous inflammation-related pathways were found to be significant in multiple clusters, including chemokine signaling pathway, IL5 signaling pathway, IL9 signaling pathway, T-cell receptor / Ras pathways, TNFα signaling pathway, mTORC1-mediated signaling, IL2/STAT5 pathway, IL17 signaling pathway, TGFβ regulation of extracellular matrix, and NF-κB in B cells mediated signaling pathways (refer to Fig. 5). Notably, TG treatment led to a significant decrease in the expression levels of several proinflammatory cytokines and chemokines that play a crucial role in neuroinflammation [6, 36]. These include chemokines (*Ccr6*, *Cx3cl1*, and *Cxcl13*), interleukins (*Il13ra1*, *Il4ra*, and *Ilf3*), and tumor necrosis factors (*Tnfaip2*, *Tnfrsf14*, and *Tnfsf11*) (Fig. 6b).

A critical finding of our study elucidates that the expression level of *Traf6*, the most divergent member of the TRAF family members, was significantly downregulated in mice hippocampus by TG treatment. PPI network analysis also suggested that TG might interfere with the Traf6-mediated signaling pathway of NF-κB **(**Fig. 6a**).** IκB kinase (IKK) complex-related kinase, *Ikbkb, was* also downregulated. Since IKK-related kinase phosphorylation has a negative effect on the canonical IKKs, they may terminate the synthesis of inflammatory mediators [37]. Furthermore, NF-κB is a group of transcription factors that are known to translocate into the nucleus and stimulate the transcription of numerous genes related to immune response, growth, and protection against apoptosis [33]. The activation of NF-κB is commonly mediated by a variety of signaling pathways that ultimately converge at the IKK complex, responsible for phosphorylating IκB and facilitating signal transduction to NF-κB [38]. Therefore, based on these findings, we postulate that TG may exert its effects on reducing inflammatory cytokine production by negatively modulating the NF-κB signaling pathway mediated by Traf6 and Ikbkb. Further exploration of this intricate mechanism holds significant promise and warrants attention.

### TG-induced changes in neurotransmitters in the hippocampus

Neurotransmitter is the major signaling molecule secreted by a neuron to communicate with other cells across a synapse to facilitate learning and memory function in the hippocampus [39, 40]. We investigated the role of TG in modulating neurotransmitters in SAMP8 mice hippocampus. *Gabra2, Adcyap1r1,* and *Adrb2* genes were upregulated after TG treatment **(**Fig. 8a**),** which might play a crucial role in GABAergic, glutamatergic, and dopaminergic synapse signaling pathways. Also, glutamate receptor subunits such as *Gria4, Grid2, and Grm5* were upregulated by TG treatment which is the main excitatory neurotransmitter in the central nervous system [41]. It is well reported that glutamatergic synapse is essential for basic brain processes, including synaptic plasticity, which is crucial for learning and memory, the formation of neuronal networks, and the CNS repair process [42]. Upregulation of glutamate receptor-related genes indicated that TG administration facilitates nervous system development, thus helping memory formation. As TG exerts a significant effect on glutamate receptors, further investigation is required to understand the mechanism of TG on glutamate neurotransmitters to facilitate learning and memory formation as well as LTP. In addition, *Igfbp6* is 1.9-fold upregulated by TG treatment which helps facilitate positive regulation of the MAPK cascade and the insulin-like growth factor receptor signaling pathway [43, 44]. Also, *Dbi* is 2.5-fold upregulated hence regulating learning and memory, glial cell proliferation, regulation of synaptic transmission, GABAergic synapse, skin development, positive regulation of lipid biosynthetic process, long-term synaptic potentiation, positive regulation of CoA-transferase activity, positive regulation of phospholipid transport related biological process. We constructed a PPI network to understand the protein–protein interactions between the target and TG (Fig. S5). Surprisingly, we noticed that when the excursive molecule; *Traf*6 was downregulated in the next step, it modulated the expression of a cluster of synapse-related genes **(**Fig. 7e**)**. Among this cluster, *Dnm1*, *Stub1*, and *Sumo1* were significantly upregulated, which are known to facilitate synapse-related pathways and help in regulating neurotransmitter release and synaptic vesicle trafficking [45, 46].

Finally, we performed a validation experiment and identified that TG treatment significantly increases DA, NA, and 5-HT levels in the hippocampus (Fig. 8 b, c, d). 5-HT interacts with its receptors and plays a role in maintaining LTP [47], deficiency of 5-HT in the hippocampus can impair memory. An increased level of neurotransmitters helps to form new memories. Further, the serotonergic system may also be involved in the modulation of synaptic plasticity and sensory input reorganization. Besides that, the neurotransmitter DA has been linked with long-term learning and memory formation. Emerging research evidence suggests that the neurotransmitter dopamine, a key brain component for long-term memory formation, is critical for motivation and also affects the hippocampus [48, 49]. Hence neurotransmitters have been linked with long-term memory formation; it can be said that TG administration improves spatial learning and memory formation by increasing neurotransmitter release and facilitating synaptic function. Therefore TG-mediated neurotransmitter level increment helps not only in learning and memory formation by suppressing proinflammatory cytokine but also helps in locomotor, facilitating behavior, feeling sensations, maintaining heart function, and information processing from the environment and other internal parts of our body, consequently helping in spatial learning and memory formation (Fig. S3).

### TG treatment mitigated synaptic functions in the mice hippocampus

An electrical impulse at a chemical synapse induces the presynaptic neuron to discharge neurotransmitters, which subsequently attach to receptors on the postsynaptic cell and influence its probability of generating an action potential. Therefore, our investigation encompassed not only examining the effects of TG on specific neurotransmitters but also exploring its impact on synapse-specific biological functions and cellular localization.

We found that synapse-specific genes that are involved in signaling pathways such as synaptic vesicle endocytosis, vesicle fusion, synaptic plasticity, LTP, etc. [50] were upregulated after TG treatment (Fig. 7d). Vesicle trafficking biomarkers such as *Vamp1, Vdac1, and Sytl2* were upregulated in the TG-treated group, indicating that it helps in vesicle trafficking and endocytosis process followed by LTP formation as the neurons communicate with each other through the synapse [44]. Synaptic plasticity is believed to underlie the processes of learning and memory retention, synaptic connections can change over time, and high-frequency signals or repeated stimulations strengthen synaptic connections repeatedly [51]. LTP occurs at most excitatory synapses all over the brain but is best studied at the glutamate synapse of the hippocampus. While TG facilitates glutamatergic synapses and upregulated synaptic gene markers, thus helping in memory formation as well as cognitive function [35]. Apart from this, we analyzed significantly enriched biological processes and found that a large number of DEGs were involved in nervous system development, neuron projection development, dendritic spine development, axon guidance, positive regulation of axon extension, chemical synaptic transmission, and synapse organization (Fig. 4A), which indicates that TG influences nervous system development, neuroprotection and improves learning and memory acquisition [52].

Moreover, many genes were involved in the synaptic biological process. Among them, 51 genes are involved in the synapse, 27 genes are involved in translation at the synapse; 27 genes are involved in translation at the pre-synapse, 27 genes are involved in translation at the post-synapse, 21 genes are involved in processing the pre-synapse, 11 genes are involved in the synaptic vesicle cycle, and 29 genes are involved in metabolism. **(**Fig. 7c**)**. As TG plays a significant role in the various synaptic biological processes, we further checked synaptic genes that are localized in synaptosomes. In this active zone, the transmission of nervous impulses is transferred from one neuron to another. Significantly upregulated synaptic DEGS were localized around the synaptosome and helped in facilizing nerve impulses, which is crucial for brain function.

Previous research indicates that chemical synapses' ultrastructure is usually asymmetric. synaptic vesicles impregnated with neurotransmitters are unidirectional, from presynaptic to postsynaptic [52]. Also, a synaptic cleft, a presynaptic element (like an axon terminal), and a postsynaptic element make up chemical synapses, which generate connections between neurons or between neurons and non-neuronal cells. Even though the molecular mechanism of learning and memory acquisition is still unclear; however, previous research has demonstrated that-synaptic elements consist of an active zone (i.e., a specific presynaptic membrane region). The high density of Ca2 + channels facilitates synaptic vesicle fuse (exocytosis), which is triggered when an action potential is received at the presynaptic terminal [53]. At the hippocampus, AMPA receptor distribution in synaptic dendritic spines during LTP formation, in the presence of TGFβ, inhibits inflammation and enhances learning and memory. We found that TG administration helps in the regulation of the MAPK cascade as well as the TGF-β signaling pathway (Fig. S4), thus reducing inflammation and increasing neurotransmitter release and, consequently, helping in memory formation [54, 55]. We checked the significantly enriched GOs of the TG-treated mice in nervous system development **(**Fig. 4**)** and found that a wide range of synapse-related BP is significantly upregulated. Therefore, our findings suggest that TG treatment may promote synaptic plasticity by upregulating synapse-related DEGs, aiding neurotransmitter release and consequently promoting spatial learning and memory formation. Further exploration of the effects of TG on synaptic functions through electrophysiological techniques or high-throughput assays in the in vitro models would therefore present a valuable avenue for future research.

## Conclusion

In summary, TG attenuated aging-induced hippocampal proinflammatory cytokines release (TNFα and IL6), potentially through regulating the Traf6-mediated NF-κB signaling pathway. Consequently, it promoted the release of neurotransmitters (DA, NA, 5-HT), which in turn facilitated memory and learning functions in aging model mice. Therefore, TG might be considered a potential supplementary medicinal compound for ameliorating cognitive aging and neuroinflammation-related CNS dysfunctions. However, further immunohistochemical, cytochemical, and proteomics analyses would strengthen and validate our findings regarding the predicted mechanism underlying the observed bioactivities of TG, as determined through transcriptomics analysis.

### Supplementary Information

Below is the link to the electronic supplementary material.

Supplementary file1 (DOCX 14 KB)

Supplementary file2 (PPTX 4237 KB)

Supplementary file3 (ZIP 92 KB)

## Data Availability

All datasets generated for this study are included in this article/Supplementary Material. Microarray data are deposited in the NCBI Gene Expression Omnibus (GEO) under accession number: GSE223398.
