# Supplementary material for: Transcriptomics and biochemical evidence of trigonelline ameliorating learning and memory decline in the senescence-accelerated mouse prone 8 (SAMP8) model by suppressing proinflammatory cytokines and elevating neurotransmitter release
Source: GeroScience. 2023 Sep 18;46(2):1671–91. doi: 10.1007/s11357-023-00919-x (PMC10828270; doi:10.1007/s11357-023-00919-x)
Supplement: Supplementary file 1 — Supplementary file1 (DOCX 14 KB) [file 11357_2023_919_MOESM1_ESM.docx]

| Neurotransmitters and  Cytokines | SAMR1  mean (SD) | SAMP8  mean (SD) | SAMP8+TG  mean (SD) | Δmean  SAMP8-SAMR1 | Δmean  SAMP8 TG-SAMP8 |
| --- | --- | --- | --- | --- | --- |
| Dopamine | 31.94 (2.19) | 20.91 (2.61) | 25.82 (2.98) | -11.03^***^ | 4.91^**^ |
| Nor-Adrenaline | 6.09 (0.75) | 3.02 (0.81) | 4.02 (0.78) | -3.07^***^ | 1.00^*^ |
| Serotonin | 32.03 (3.32) | 13.66 (1.94) | 18.25 (2.09) | -18.37^***^ | 4.59^**^ |
| BDNF | 9.66 (1.33) | 1.79 (0.39) | 2.48 (0.94) | -7.87^***^ | 0.69 ns |
| TNFα | 27.83 (2.73) | 57.42(6.30) | 41.35(6.82) | 29.59^***^ | -16.07^***^ |
| IL6 | 248.93 (16.04) | 294 .63(14.44) | 274.05 (19.64) | 45.70^***^ | -20.58^*^ |

Supplementary Table 1

All the ELISA experiment Data

Statistical analysis was performed by using one-way ANOVA followed by Fisher’s LSD test: *P<0.05; **P<0.01; ***P<0.001.
