# Supplementary material for: Transcriptomics and biochemical evidence of trigonelline ameliorating learning and memory decline in the senescence-accelerated mouse prone 8 (SAMP8) model by suppressing proinflammatory cytokines and elevating neurotransmitter release
Source: GeroScience. 2023 Sep 18;46(2):1671–91. doi: 10.1007/s11357-023-00919-x (PMC10828270; doi:10.1007/s11357-023-00919-x)
Supplement: Supplementary file 2 — Supplementary file2 (PPTX 4237 KB) [file 11357_2023_919_MOESM2_ESM.pptx]

## Slide 1
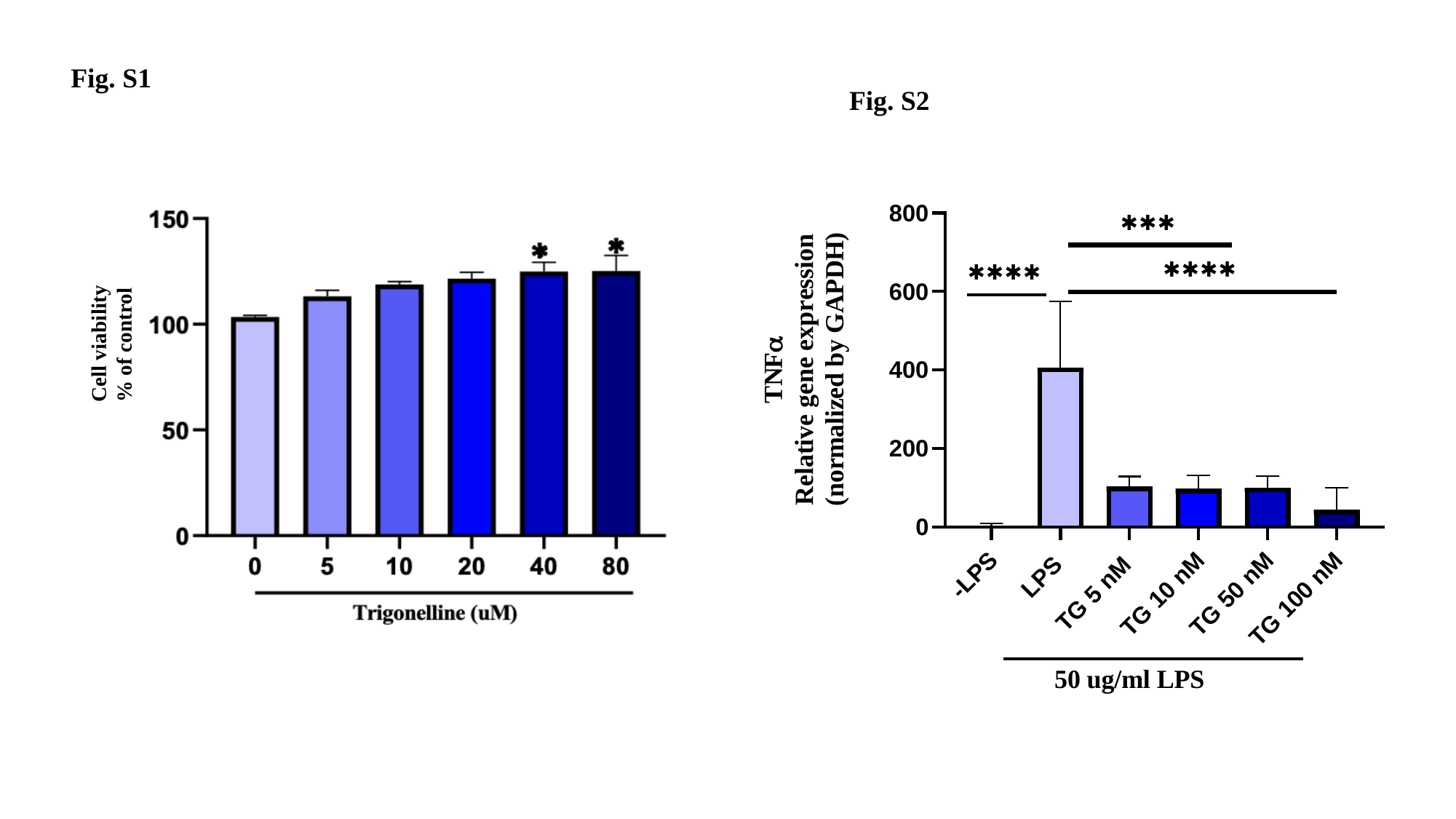

Fig. S1
Fig. S2
Cell viability
% of control

## Slide 2
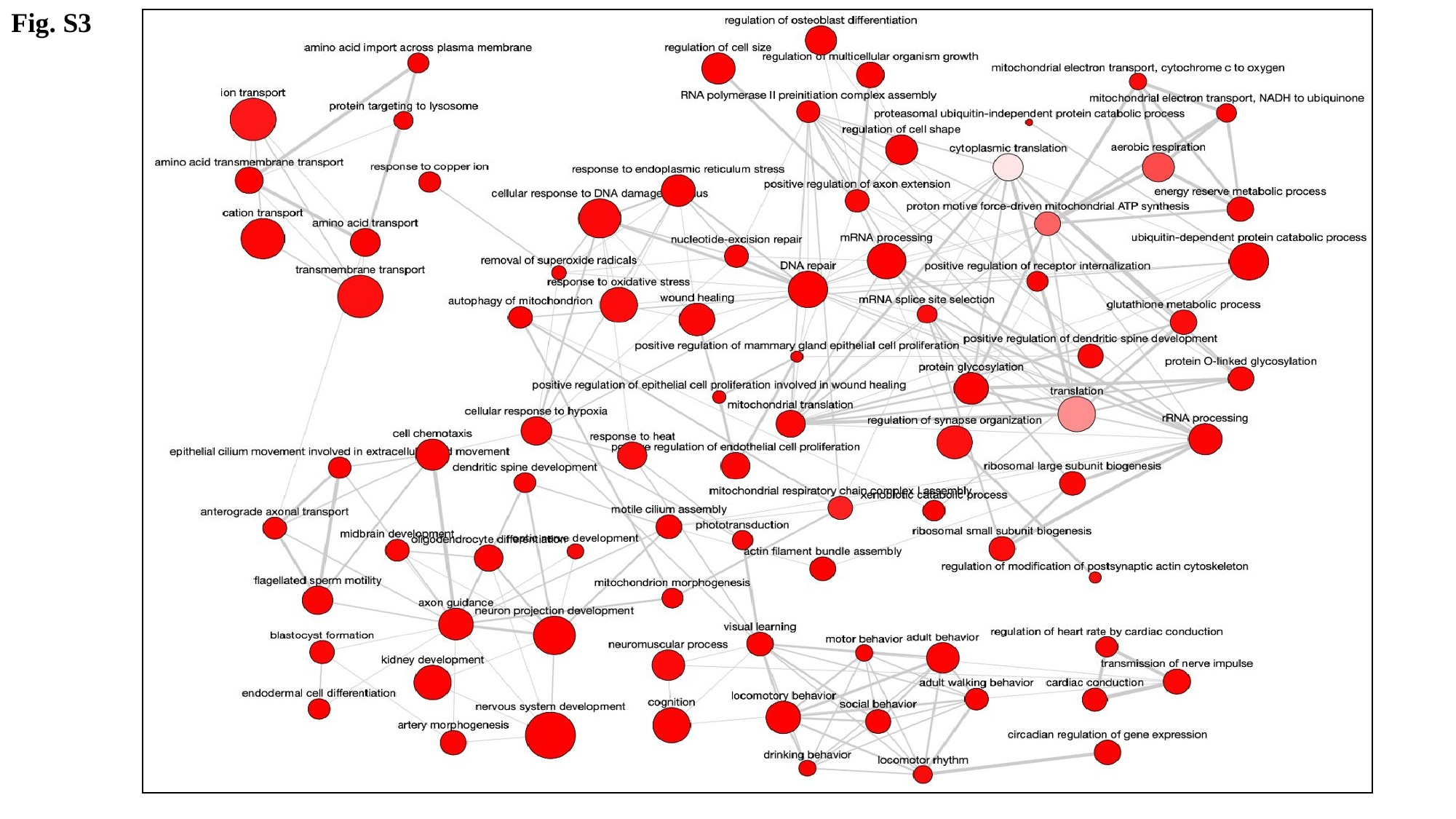

Fig. S3

## Slide 3
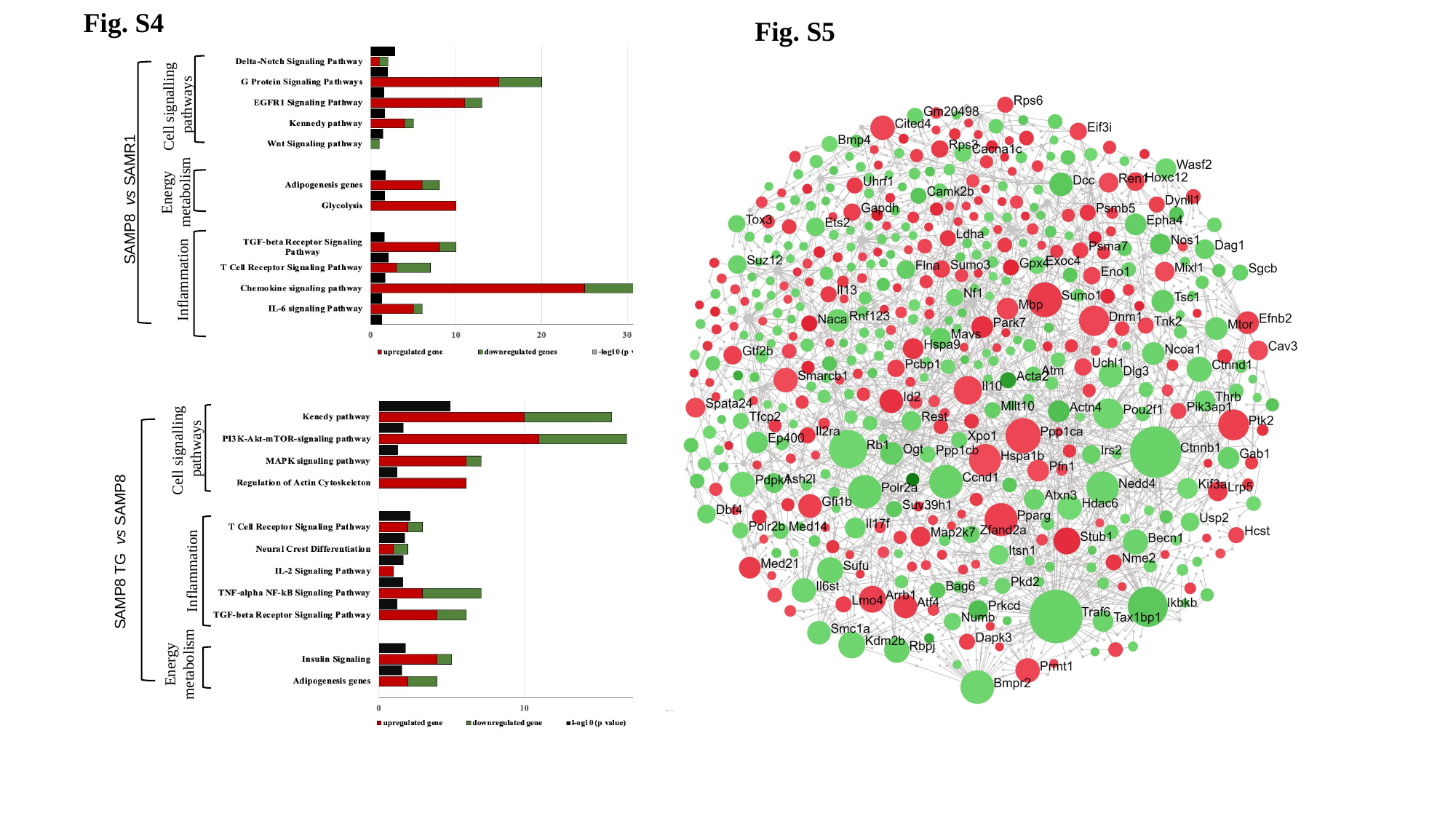

Fig. S4
Fig. S5
Cell signalling
pathways
Energy metabolism
Inflammation
SAMP8 vs SAMR1
Inflammation
Energy metabolism
Cell signalling
pathways
SAMP8 TG vs SAMP8
